# Sexually Dimorphic Expression of *vasa* Isoforms in the Tongue Sole (*Cynoglossus semilaevis*)

**DOI:** 10.1371/journal.pone.0093380

**Published:** 2014-03-26

**Authors:** Zhongkai Wang, Jinning Gao, Huayu Song, Xiaomeng Wu, Yan Sun, Jie Qi, Haiyang Yu, Zhigang Wang, Quanqi Zhang

**Affiliations:** Key Laboratory of Marine Genetics and Breeding (MGB), Ministry of Education, College of Marine Life Sciences, Ocean University of China, Qingdao, China; Temasek Life Sciences Laboratory, Singapore

## Abstract

The *vasa* gene encodes an ATP-dependent RNA helicase of the DEAD box protein family that functions in a broad range of molecular events involving duplex RNA. In most species, the germline specific expression of *vasa* becomes a molecular marker widely used in the visualization and labeling of primordial germ cells (PGCs) and a tool in surrogate broodstock production through PGC transplantation. The *vasa* gene from tongue sole (*Cynoglossus semilaevis*) was characterized to promote the development of genetic breeding techniques in this species. Three *C. semilaevis vasa* transcripts were isolated, namely *vas-l*, *vas-m*, and *vas-s*. Quantitative real-time PCR results showed that *C. semilaevis vasa* transcripts were prevalently expressed in gonads, with very weak expression of *vas-s* in other tissues. Embryonic development expression profiles revealed the onset of zygotic transcription of *vasa* mRNAs and the maternal deposit of the three transcripts. The genetic ZW female juvenile fish was discriminated from genetic ZZ males by a pair of female specific primers. Only the expression of *vas-s* can be observed in both sexes during early gonadal differentiation. Before PGCs started mitosis, there was sexually dimorphic expression of *vas-s* with the ovary showing higher levels and downward trend. The results demonstrated the benefits of *vasa* as a germline specific marker for PGCs during embryonic development and gonadal differentiation. This study lays the groundwork for further application of *C. semilaevis* PGCs in fish breeding.

## Introduction

DEAD (Asp–Glu–Ala–Asp) box protein families are ATP-dependent RNA helicases present in almost all organisms. These proteins have significant functions in RNA metabolism and are associated with processes involving RNA from transcription to degradation [1]. DEAD-box proteins comprised three subfamilies: VASA, PL10, and P68. The *vasa* gene was thought to arise from the duplication of a PL10-related gene prior to the appearance of sponges, but following the diversion of fungi and plants [2]. Since the first isolation of the *Drosophila vasa* gene [3], *vasa*-like genes have been identified from invertebrates (e.g., hydra [2], planarian [4], nematode [5], and ascidian [6]) to vertebrates (e.g., Xenopus [7], chicken [8], mouse [9], and human [10]). With these observations, recent studies have revealed that the *vasa* gene and its products are restricted to the germ cell lineage in most species. Extragonadal *vasa* expression was also reported in teleosts, such as rainbow trout [11], European sea bass [12], and Senegalese sole [13].

Studies on teleosts have investigated on the *vasa* expression patterns during the embryogenesis and gonadal differentiation. *Vasa* isoforms were characterized in several teleosts such as zebrafish [14], [15], tilapia [16], rare minnow [17], Japanese flounder [18], and Senegalese sole [13]. The expression profiles of different *vasa* transcripts were distinguished during sex differentiation in zebrafish [19], tilapia [16], and Senegalese sole [13]. This distinction implies the functions of these isoforms in germline development and the significance of homolog characterizations.

The *vasa* gene is a molecular marker widely used in visualizing and labeling of primordial germ cells (PGCs). The stable labeling of these cells in teleosts is achieved through the specific expression of green fluorescence protein (GFP) driven by the *vasa* promoter. Transgenic fish lines have been reported only in model fish (e.g., zebrafish [20] and medaka [21]) and aquaculture species (e.g., rainbow trout [22]). PGCs are the progenitor cells of germ cell lineage responsible for genetic transmission to the next generation, which allow their applications in fish bioengineering, such as cryopreservation of the genetic resources [23], [24] and transplantation for surrogate broodstock production [25]–[28]. These studies suggest the bioengineering potential of the *vasa* gene as a tool in fish breeding.

Tongue sole (*Cynoglossus semilaevis*) is an economically important marine fish in China. To date, only wild *C. semilaevis* broodstocks are used to produce robust eggs, thereby hampering the development of genetic breeding techniques. PGC transplantation provides a novel approach to generate viable offspring by surrogate breeding. We identified *vasa* transcripts in *C. semilaevis* to label and isolate their PGCs. We cloned and characterized the full length of three *C. semilaevis vasa* isoforms for the first time. We quantified the distribution patterns of each *vasa* transcript in adult tissues and the expression profiles during embryogenesis and gonadal differentiation. The results will facilitate further studies on labeling, isolation, and transplantation of PGCs as well as promote the progress of breeding techniques in this species.

## Methods and Materials

### Ethics Statement

*C. semilaevis* (*Pleuronectiformes*) samples were collected from local aquatic farms with permission from the local government of Yantai, Shandong, China. The samples were handled in accordance with the guidelines and regulations established by the Ocean University of China and the local government of Yantai.

### Fish

All fish and embryos were collected from a commercial farm in Yantai, Shandong Province, China. All procedures complied with and were approved by the Institutional Animal Care and Use Committee of the Ocean University of China.

Six randomly selected healthy 2-year-old adults (three females and three males) were dissected. Tissues from the muscle, gill, heart, intestine, brain, kidney, liver, spleen, and gonad were collected.

Artificially fertilized eggs were incubated at 21±1°C in the hatching tanks with an open recirculation water system and sufficient air supply. Unfertilized eggs and embryos of different developmental stages (n = 50; 1-, 2-, 8-, and 16-cell, morula, high and low blastula, early and late gastrula, Kupffer's vesicle, neurula, somitogenesis, and hatching) were collected. Each sample was collected in triplicate.

Juvenile fish cultivated at 23°C during early gonadal differentiation [n = 10 per group; total length (TL)  =  20±1, 25±1, 30±1, 35±1, 40±1, 45±1, 50±1, and 55±1 mm] were dissected. Isolating gonads from juveniles with TL≤60 mm is difficult, so we collected the whole abdomen that contained the gonadal anlagen by removing the head as well as most of the muscular dorsal and ventral parts of the fish. The muscle tissues of each fish were also collected.

Each sample was collected in triplicate. All samples were immediately frozen using liquid nitrogen and stored at −80°C for total RNA or genomic DNA preparation.

### RNA and genomic DNA extraction

Total RNA was extracted using Trizol Reagent (Invitrogen, CA, USA) according to the manufacturer's protocol, treated with RNase-free DNase I (TaKaRa, Dalian, China) to degrade genomic DNA, and then frozen at −80°C. cDNA synthesis was performed with 1 μg total RNA and random hexamer primers using Reverse Transcriptase M-MLV (RNase H^−^) Kit (TaKaRa, Dalian, China) following the manufacturer's instructions. Genomic DNA was isolated from the muscle tissue using the traditional phenol/chloroform extraction method.

The quality and quantity of the RNA and DNA were evaluated by 1.5% agarose gel electrophoresis and spectrophotometry using NanoPhotometer Pearl (Implen, Munich, Germany).

### Molecular cloning of *vasa* gene

*De novo* transcriptome sequencing and characterization for *C. semilaevis* were performed in our laboratory. The raw reads were submitted to SRA Databases in NCBI (Accession number: SRX257138). A total of 749,954 reads generated using a single 454 sequencing run in one full PicoTiter plate were assembled into 62,632 contigs with a ten-fold average sequencing coverage.

Two contigs were screened from the *C. semilaevis* transcriptome through sequence homology analysis. A pair of scaf-FW/RV primers (Table S1) was designed separately based on the contigs. PCR amplification was performed to link the two contig sequences under the following conditions: initial denaturation at 95°C for 5 min, followed by 30 cycles at 95°C for 30 s, at 60°C for 30 s, and at 72°C for 1 min; and a final extension at 72°C for 10 min.

The 5′ and 3′ RACE were performed to isolate the full-length cDNA of *vasa* from the ovary tissue using SMART RACE cDNA Amplification Kit (Clontech, CA, USA) according to manufacturer's protocol. Gene-specific primers (GSPs) were designed based on the known cDNA sequence. For the 5′ RACE, the GSPs were 5′ race1 and 5′ race2 (Table S1); for the 3′ RACE, the GSPs were 3′ race1 and 3′ race2 (Table S1). PCR was conducted according to the SMART RACE amplification methods. The full length of the *vasa* cDNA sequence was assembled with the sequenced PCR products using software suite Lasergene v7.0 (DNASTAR, WI, USA).

Genomic sequence of *vasa* was cloned through PCR amplification using LA Taq (TaKaRa, Dalian, China) with specific primers vasa-5′ FW and vasa-3′ RV (Table S1) designed based on the potential 5′ and 3′ UTR of the obtained *vasa* cDNA sequence. Subsequently, the genomic 5′ and 3′ *vasa* flanking sequences were amplified using the Genome Walking Kit (TaKaRa, Dalian, China); the primers 5′ GSP1/5′ GSP2/5′ GSP3 and 3′ GSP1/3′ GSP2/3′ GSP3 (Table S1) were designed according to the instructions. The PCR conditions likewise followed supplied instructions in the kit.

All PCR products were separated on 1.5% agarose gel electrophoresis, purified using the Zymoclean Gel DNA Recovery Kit (Zymo Research, CA, USA), cloned into pMD-18T Vector (TaKaRa, Dalian, China), and sequenced.

### Phylogenetic analysis of *vasa* products

Homologous nucleotide and protein sequences were confirmed through the BLAST search at NCBI (http://www.ncbi.nlm.gov/blast). Multiple sequence alignments were conducted by ClustalX 2.1 (http://www.clustal.org/clustal2/). The phylogenetic tree was constructed using MEGA5 [29] with neighbor-joining method [30]. The branching reliability was tested via bootstrap resampling with 1,000 pseudoreplicates.

### Tissue distribution pattern of *vasa* isoforms by quantitative real-time PCR (qRT–PCR)

Three specific primer pairs (vasaL-FW/RV, vasaM-FW/RV, vasaS-FW/RV; Table S1) were designed based on the characteristics of *vas-l*, *vas-m,* and *vas-s*. Pre-experiment was conducted to confirm a single cDNA PCR product and avoid the amplification of genomic DNA. Specific PCR products were verified through sequencing.

The evaluation of eight housekeeping genes as candidate references for gene expression analysis in *C. semilaevis* was assayed in our laboratory (unpublished). *18S rRNA* was the most stable gene in the different tissues. Therefore, relative expression was determined using the *18S rRNA* as the reference gene.

Total RNA was extracted from the adult tissues and cDNA synthesis was performed. Three biological replicates of each sample were analyzed, with each sample ran in triplicate.

qRT–PCR was performed in a 20 μl solution containing 20 ng template cDNA and SYBR Premix Ex Taq II (TaKaRa, Dalian, China) via LightCycler480 (Roche Applied Science, Mannheim, Germany) at 95°C (5 min) for pre-incubation, followed by 45 cycles at 95°C (15 s) and 60°C (45 s); finally, the melting curve was analyzed to detect single amplification. The accumulation of fluorescent signal from SYBR Green I was recorded at 60°C (45 s) phase during each cycle under the control of LightCycler480 Software 1.5. Negative control (no-template reaction) was always included.

Relative quantification for the target *vasa* gene that is expressed as fold variation over the reference gene *18S rRNA* was calculated by the 2−ΔΔCt comparative Ct method.

### Histology

Gonad samples (ovary and testis) for histological observation were fixed in Bouin's solution for 24 h and then stored in 70% ethanol at 4°C.

The fixed gonads were dehydrated in an ascending ethanol series, cleared with xylene, and embedded in paraffin wax. The 6 μm-thick sections were stained with hematoxylin and eosin (H&E) and observed under a Nikon Eclipse Ti-U microscope (Nikon, Tokyo, Japan).

### In situ hybridization (ISH)

The gonad samples used for ISH were fixed immediately in 4% paraformaldehyde–PBS (4% PFA) overnight at 4°C, dehydrated in gradients of increasing methanol and stored in 100% methanol at −20°C.

ISH of *vasa* expression in the gonads was performed using a 364 bp probe spanning the 3′ UTR of the *vasa* cDNA (Table S1). DIG-labeled RNA sense and anti-sense probes were synthesized using the DIG RNA Labeling Kit (SP6/T7) (Roche, Mannheim, Germany) according to the manufacturer's instructions. ISH on paraffin sections of the gonads was performed [31], [32].

### Ontogenic expression patterns of *vasa* isoforms by qRT–PCR

Expression patterns of *vasa* isoforms during embryogenesis was analyzed by relative qRT–PCR. Total RNA was prepared from the embryo samples collected at different developmental stages. Housekeeping gene *B2M* was the most stable gene during embryo development among the eight candidate reference genes and used as the reference gene.

### Expression pattern of *vasa* isoforms during early gonadal differentiation by qRT–PCR

Genomic DNA was isolated from the juvenile fish muscle. Subsequently, the genetic sex of each fish was identified by PCR, which was conducted using control and female-specific primers developed by our laboratory [33]. The PCR products were separated on 1.5% agarose gel. Female and male individuals were discriminated from each other by the existence of a female specific band. Then, 3 female and 3 male juvenile fish were selected from each group using the total length (TL), and the fish were used to analyze the expression pattern of *vasa* isoforms during early gonadal differentiation by relative qRT–PCR. Housekeeping gene *RPL17* was the most stable during larval development among the eight candidate reference genes and used as the reference gene.

### Statistical analysis

qRT–PCR data were statistically analyzed by one-way ANOVA followed by a Tukey's post-hoc test using SPSS 20.0 (SPSS, IL, USA), and p<0.05 denotes statistically significant difference.

## Results

### Molecular characterization of three *vasa* isoforms

A longer 1232 bp cDNA fragment containing the two contigs that was screened from the *C. semilaevis* transcriptome was obtained using scaf-FW/RV primers. The remaining parts of *vasa* were cloned through 5′ and 3′ RACE. Three types of 5′ extremities were identified from the sequenced 5′ RACE products. The three *vasa* isoforms were assembled with the cDNA fragments.

The longest isoform was the 2495-bp long *vas-l* (GenBank: KF856010), which contained an open reading frame (ORF) of 2046 bp and encoded 681 amino acid residues (Figure S1). The shortest was the 2441-bp long *vas-s* (GenBank: KF856012), which contained an ORF of 1992 bp and encoded 663 amino acid residues (Figure S1). The nucleotide sequence of the 2468-bp long *vas-m* (GenBank: KF856011) contained an ORF of 2019 bp ORF and encodes 672 amino acid residues (Figure S1).

### Genomic and phylogenetic analysis of *vasa*

A 6388 bp genomic *vasa* fragment (GenBank: KF856013) was cloned based on the *vasa* cDNA sequence. Only the single genomic 5′ flanking sequence (GenBank: KF876914) or 3′ flanking sequence (GenBank: KF876915) of *vasa* was obtained through genome walking.

**Figure 1:**
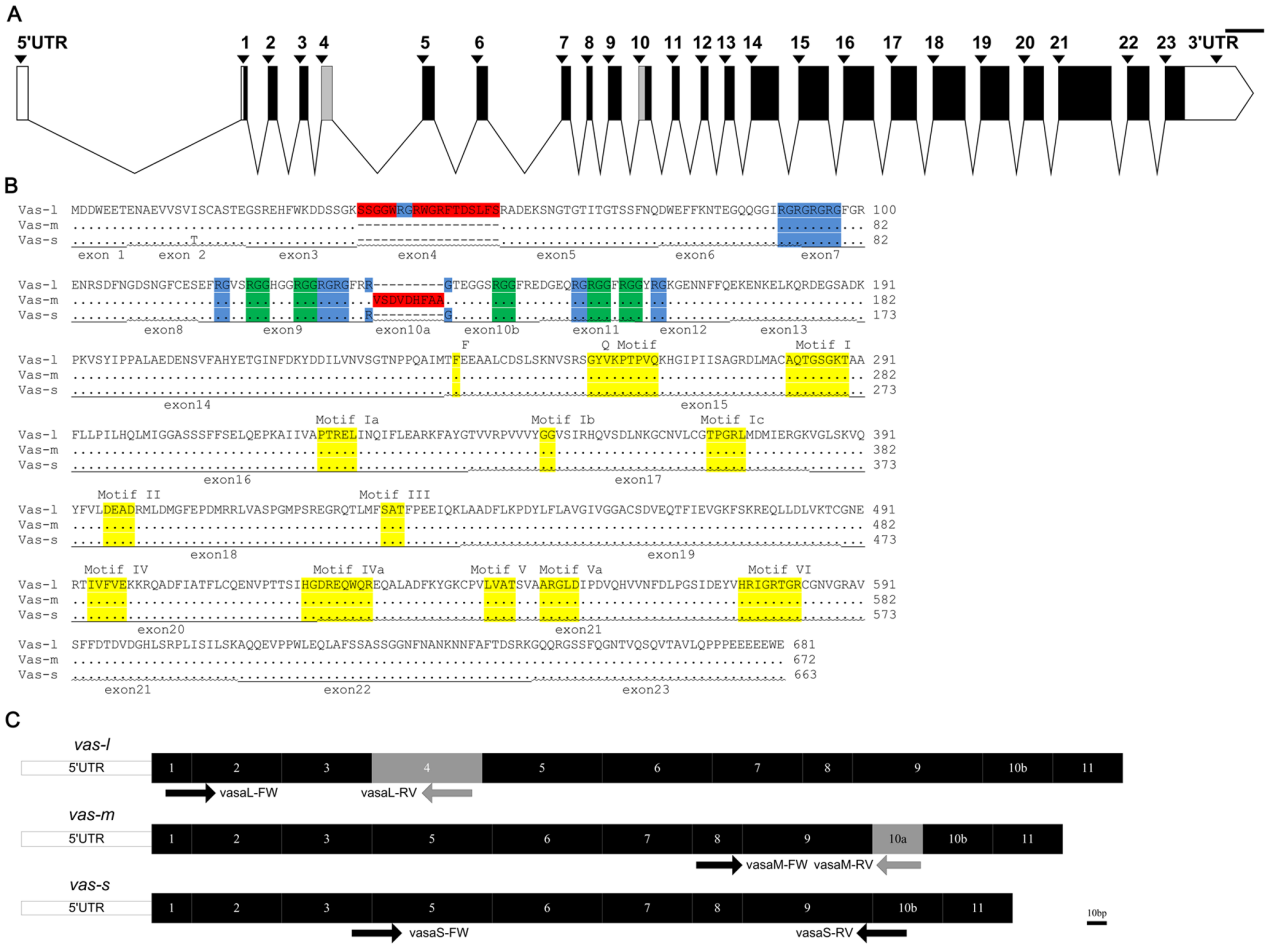
Schematic structure of *vasa* gene and its products. (A) A graphical representation of *vasa* genomic structure was drawn. Exons were shown as boxes and numbered above the schematic (protein coding region in the black or grey box; UTR in the open box); introns were shown as fold lines. Exons 4 and 10a were shown as grey boxes. Scale bar = 200 bp. (B) Multiple sequence alignments of the three Vasa proteins were performed and the major motif locations were marked under the sequences. The motifs encoded by either exon 4 or 10a were boxed in red background. The motifs conserved among Vasa characteristic homologs of the DEAD-box proteins were boxed in yellow background. These motifs were encoded by exons 15 to 21. The RGG and RG repeats at the N-terminal regions were boxed in green and blue backgrounds and encoded by the exons 4 to 12. Given the sequence differences, Vas-l, Vas-m, and Vas-s had 11, 9 and 10 RG repeats, respectively. (C) The 5′ parts of the three *vasa* isoforms were graphically presented. The position of the specific primers used to clone the *vasa* isoforms was shown under the corresponding transcript. Exon 4 uniquely encoded the amino acids in *vas-l* transcript; exon 10a functioned as a part of CDS only in *vas-m* transcript. Scale bar = 10 bp.

Comparison of the three *vasa* cDNA sequences with the genomic sequence revealed a possible genomic structure of *vasa* gene (Figure 1A). The *vasa* gene contained up to 23 exons, and the 5′ UTR was separated by the first and the longest intron.

The deduced amino acid sequence alignments of the three variants were conducted by ClustalX 2.1; the motif locations were shown in terms of the genomic structure of the *vasa* gene (Figure 1B). They shared significant similarities with the same highly conserved motifs characteristics of the DEAD box protein family. The three isoforms also contained the same five arginine–glycine–glycine (RGG) motifs and the different numbers of arginine–glycine (RG) motifs resulting from the sequence differences at N-terminal regions (Figure 1B).

Based on the genomic structure and the motif locations, we divided exon 10 into two parts, i.e., exons 10a and 10b (Figure 1B). Exon 10a was a part of coding sequence (CDS) only in *vas-m* transcript, but exon 10b was found in all three transcripts (Figure 1C). Exon 4 also uniquely functioned in encoding the amino acids in *vas-l* transcript (Figure 1C).

**Figure 2:**
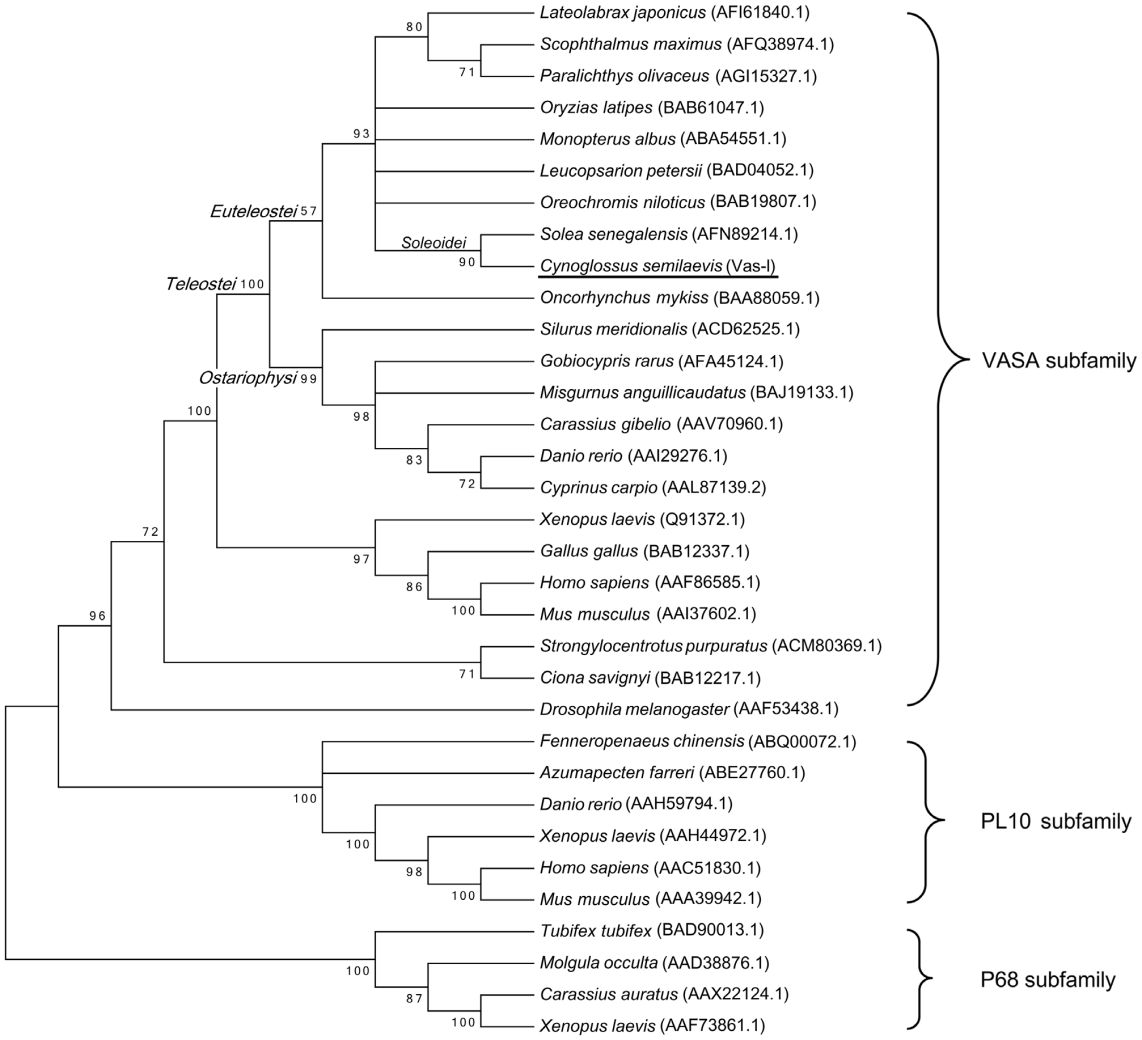
Molecular phylogenetic analysis of *vasa* products by neighbor-joining method. The bootstrap consensus tree (1,000 replicates) represented the evolutionary history of the analyzed taxa. The evolutionary distances were computed using the Poisson correction method. The bootstrap value (%) of 1,000 replicates was shown next to the branches. Branches corresponding to partitions reproduced in less than 50% of the bootstrap replicates were collapsed. The GenBank accession numbers were shown in brackets after each species. Vas-l was clustered in the VASA subfamily in the teleost branch with the Vasa homologue from *S. senegalensis* (*Soleoidei*).

Phylogenetic analysis of the DEAD-box protein family revealed that Vas-l segregated with the VASA subfamily and did not cluster with the related proteins P68 and PL10 (Figure 2). Within the VASA clade of sequences, Vas-l clustered in the teleost branch with VASA homolog from another *Soleoidei*, *Solea senegalensis*.

### Tissue distribution pattern of *vasa* transcripts detected by qRT–PCR

**Figure 3:**
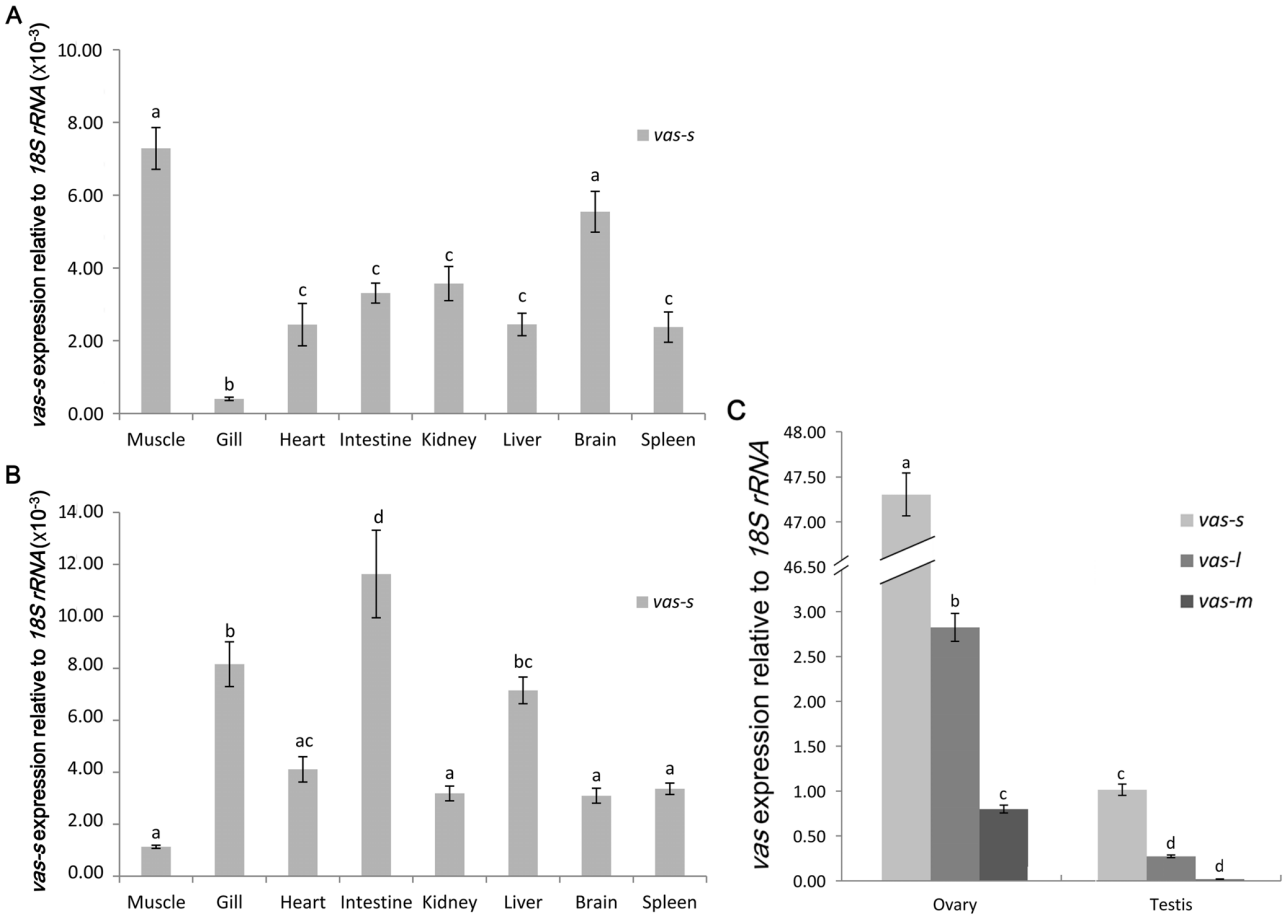
Tissue distribution pattern of *vasa* transcripts. (A) The relative expression levels of *vas-s* in the adult tissues from males were analyzed. The relative expression level of *vas-s* in the testis was used as calibrator. (B) Relative expression levels of *vas-s* in the adult tissues from females were analyzed. The relative expression level of *vas-s* in the ovary was used as calibrator. (C) The relative expression levels of *vasa* transcripts in the testis were compared with that in the ovary. The relative expression level of *vas-s* in testis was used as calibrator. The variance in the relative expression was represented as a ratio (the amount of *vasa* mRNA normalized to the corresponding *18S rRNA* values). Data were shown as mean±SEM (n = 3). Values with different superscripts indicated statistical significance (p<0.05), which were calculated via one-way ANOVA.

Analyzing the expression of the three *vasa* isoforms in *C. semilaevis* by qRT–PCR revealed that only *vas-s* could be detected in the tissues, except in the gonads. Moreover, the expression level was about three orders of magnitude lower than those in the gonads (Figure 3A and 3B). The expression level of *vas-s* was predominant, whereas *vas-m* was almost undetected compared with that of *vas-s*. The expression level of *vas-l* was considerable at approximately 25% of that of *vas-s*. The relative expression level of *vasa* mRNA was much higher in the ovary compared with that in the testis (Figure 3C).

### Localization of *vasa* mRNA*-*positive cells in the gonads by ISH

**Figure 4:**
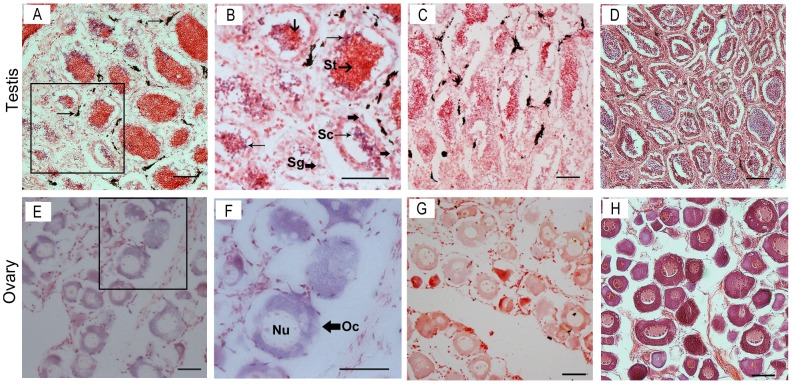
Expression of *vasa* mRNA in the gonads analyzed by in situ hybridization and histology. The *vasa* mRNA-positive cells (A, B, E, and F) were stained as purple or blue, whereas the negative control with sense probe hybridization (C and G) was unstained. Others were stained with H&E (D and H). The box indicates the area magnified in the next frame (A, C, D, E, G, and H: 20×; B and F: 40×). The *vasa* mRNA transcripts were observed in the spermatogonia and spermatocytes, but no signals were detected in the spermatids in the testis (B). The black substance observed adjacent to the seminiferous lobules (arrow, A) was a sediment in the testis. Noticeable positive signals were exhibited in the cytoplasm of oocytes (F). Abbreviations: Sg, spermatogonia; Sc, spermatocytes; St, spermatids; Oc, oocytes; Nu, nucleus. Scale bars = 50 μm.

The expression and distribution of *vasa* during oogenesis and spermatogenesis were detected using ISH with DIG labeled anti-sense RNA probe. In the testis, histological observation revealed that the cells mainly comprised spermatocytes and spermatids. ISH results revealed that the *vasa* mRNA-positive signals were strong in the spermatocytes and were located at the peripheral region of the cysts. By contrast, the signals in spermatogonia were weaker than those in spermatocytes. No signal was detected in the spermatids and testicular somatic cells (Figure 4). The ovary mainly comprised stage II oocytes. The strong signal of *vasa* mRNA was uniformly distributed throughout the cytoplasm of the oocytes. No signal was likewise detected in the somatic cells.

### Expression pattern of *vasa* at different stages during embryonic development

**Figure 5:**
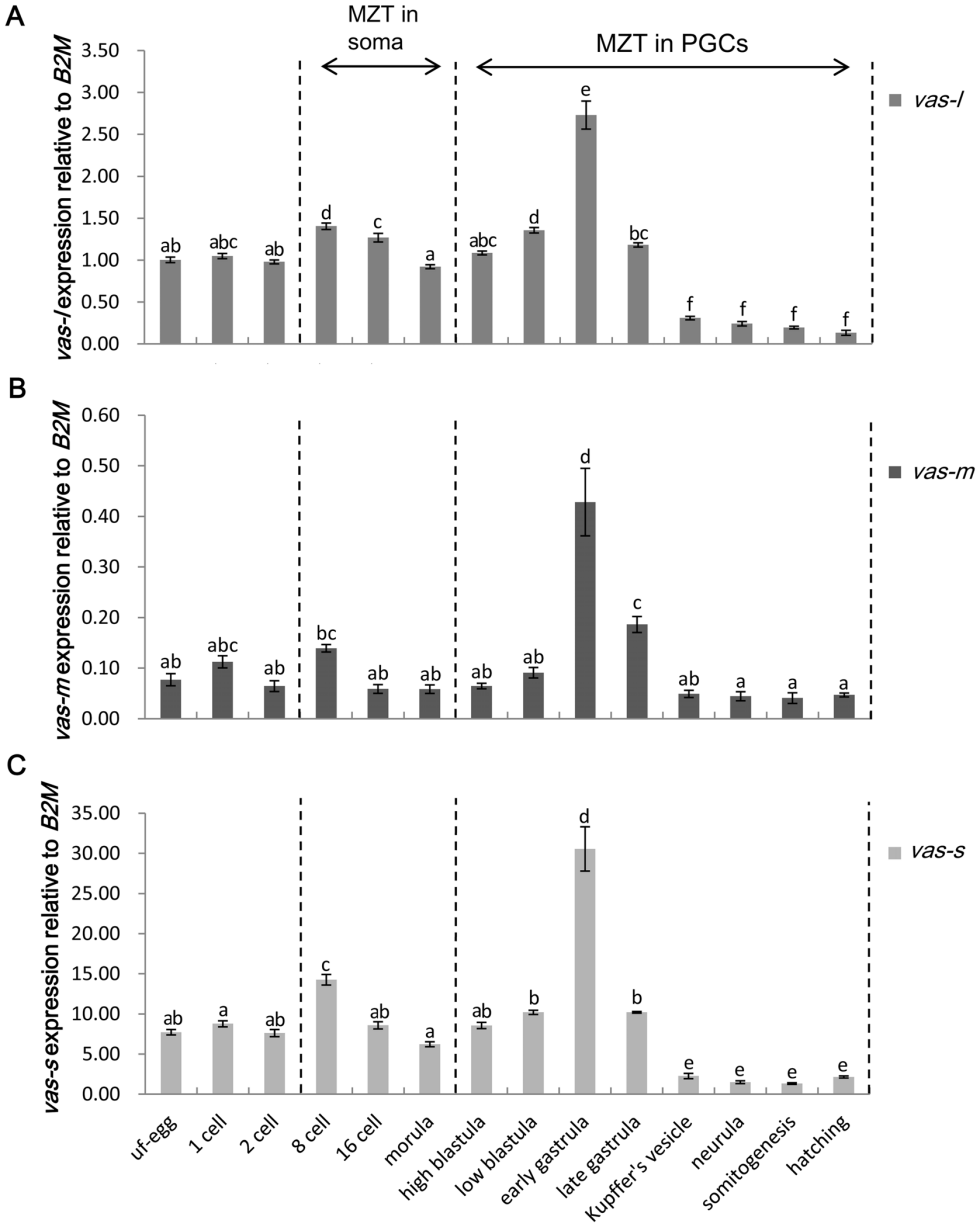
Expression pattern of *vasa* transcripts at different stages during embryonic development. Relative expression levels of *vas-l* (A), *vas-m* (B), and *vas-s* (C) during embryonic development were examined. Abbreviations: uf-egg, unfertilized egg; MZT, maternal-to-zygotic transition. The relative expression level of *vas-l* in uf-egg was used as calibrator. The variance of this expression was represented as a ratio (the amount of *vasa* mRNA normalized to the corresponding *B2M* values). Data were shown as mean±SEM (n = 3). Values with different superscripts indicated statistical significance (p<0.05), which were calculated via one-way ANOVA.

The expression patterns of three *vasa* isoforms during embryonic development at 14 different stages were monitored by qRT–PCR. Three transcripts were detected in all stages, and their expression patterns were similar except for the expression levels relative to *B2M*. In all stages, *vas-s* expression level was also predominant similar to those in the gonads. The relative expression of all isoforms fluctuated in the first six stages with the highest level at the 8-cell stage. Following the high blastula stage, the expression levels of all isoforms increased, quickly reached the peak values at the early gastrula stage, and lowered sharply at the late gastrula stage. Thereafter, the expression was maintained at a low level until the hatching stage (Figure 5).

### Expression pattern of *vasa* isoforms during early gonadal differentiation by qRT–PCR

**Figure 6:**
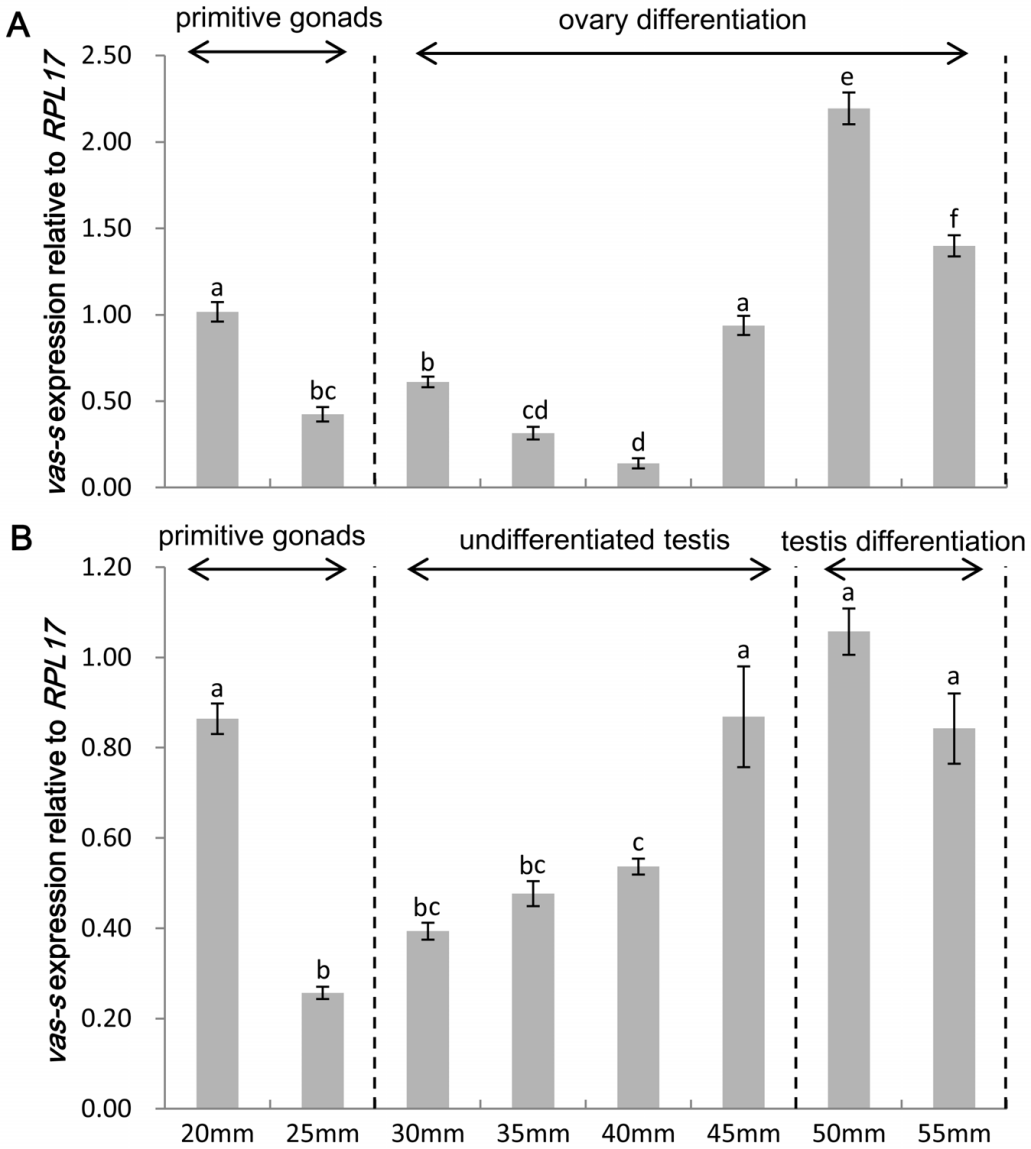
Expression pattern of *vas-s* during early gonadal differentiation. Relative expression levels of *vas-s* during gonadal differentiation in females (A) and males (B) were shown. The relative expression level of *vas-s* at the initial stage in the ovary was used as calibrator. The stages were divided according to the histological observation of *C. semilaevis* gonadal differentiation [75]. Primitive gonads appeared in juveniles having TL≤25 mm with the development of gonadal anlagen and the rapid proliferation of PGCs. The female juveniles entered into gonadal differentiation earlier than the males, and histological gonadal differentiation was first detected until females TL≥40 mm. This differentiation was signaled by the appearance of oogonia in the ovary. The testis remained undifferentiated until males TL≥50 mm with the rapid proliferation of spermatogonium and somatic cells forming seminiferous duct anlagen. The variance of this expression was represented as a ratio (the amount of *vas-s* mRNA normalized to the corresponding *RPL17* values). Data were shown as mean±SEM (n = 3). Values with different superscripts indicated statistical significance (p<0.05), which were calculated via one-way ANOVA.

Only the expression of *vas-s* could be detected and showed sexually dimorphic during early gonadal differentiation. In female juveniles, the *vas-s* expression decreased with the TL from 20 mm to 25 mm. When gonadal differentiation started in the ovary, *vas-s* expression level continued to descend from TL = 30 mm to TL = 40 mm, at which it reached the lowest value. Then, *vas-s* expression level increased and quickly reached the peak at TL = 50 mm (Figure 6A). In male juveniles, the *vas-s* expression also declined from TL = 20 mm to TL = 25 mm. The expression increased steadily until TL = 50 mm, the point at which testis differentiation occurred (Figure 6B). The *vas-s* expression level in both sexes slightly decreased from TL = 50 mm to TL = 55 mm. In addition, the expression levels of *vas-s* in females were higher than those in males, except at TL = 40 mm.

## Discussion

In this study, three isoforms of *vasa* homologs were identified in *C. semilaevis*. Multiple sequence alignments revealed that although slight sequence differences existed at the N-terminal regions, all deduced amino acid sequences of *vasa* variants contained the same conserved motifs characteristics of DEAD box proteins. Previous studies have implied that RG and RGG motifs functioned in RNA binding [34], [35] and subcellular protein localization [36]. Q motif, and Motifs I, II and VI were ATP binding and hydrolysis motifs [37]–[47]. Motifs Ia, Ib, Ic, IV, IVa, and V worked as RNA binding motifs [40], [47]–[49]. Motifs III and Va were intramolecular interactions motifs [40], [43], [50]–[52]. These motifs interact closely with each other to form an ATPase active site and a nucleic acid binding pocket so that the protein can catalyze hydrolysis of ATP and unwind RNA duplexes through non-processive, local strand separation [53], [54]. The data strongly suggest that all the isolated isoforms encode DEAD-box proteins possessing ATP-dependent RNA helicase activities.

VASA, PL10 and P68 subfamilies are important DEAD-box protein family members. Phylogenetic analysis of *vasa* proteins involved other DEAD-box proteins from both vertebrates and invertebrates. The results revealed that the *vasa* proteins most closely resemble the VASA subfamily instead of the other DEAD-box protein family members, such as the P68 and PL10 subfamilies. The phylogenetic tree showed that the *vasa* proteins are clustered with the VASA homologue from *S. senegalensis* (*Soleoidei*; *Euteleostei* sub-branch). Therefore, it further confirmed that the sequences obtained in this study are *C. semilaevis vasa*-like genes.

Three *vasa* isoforms were currently cloned in *C. semilaevis*. In addition, two *vasa* isoforms have been isolated in tilapia [16], rare minnow [17], and zebrafish [19]. In zebrafish, the long isoform contains one more exon 3 (length, 48 bp) resembling the exon 4 (length, 54 bp), which is uniquely located in *vas-l*. More than three isoforms are found in other flatfishes, such as Senegalese sole [13] and Japanese flounder [18]. The three variants in this study are only different in the 5′ extremities at exons 4 and 10, similar to the ten isoforms detected in Japanese flounder that vary in the 5′ extremities from exons 1 to 10 with the same 3′ extremities. By contrast, the four *vasa* isoforms found in Senegalese sole vary in the 5′ and 3′extremities.

Comparing the genomic locus of *vasa* in *C. semilaevis* with those in Japanese flounder [18] and zebrafish [19] revealed that all *vasa* genes contain more than twenty exons with the 5′ UTR separated by the first intron. Meanwhile, the first intron in *C. semilaevis* was the longest (length, 1101 bp). The 5′ UTR is directly connected to the first exon and regulates gene expression. We conclude that the first intron regulates the *vasa* gene transcription. We can further focus on the potential transcription factor binding sites at the intron or the non-coding RNA produced by this intron.

Given that only one *vasa* gene locus exists in the zebrafish genome, the two *vasa* isoforms are attributed to the alternative splicing [14], [15], [19], [20]. On the contrary, three *vasa* gene loci were found in the tilapia genome [55]. In this case, further studies are necessary to determine how the tilapia *vasa* isoforms are produced. On the one hand, the genome walking results in this study revealed that no sequence polymorphism existed at the 5′ or 3′ flanking of the *vasa* gene. On the other hand, only one PCR product contained exon 4 or 10 in three males and three females (Figure S2). This result suggested that no sequence differences exist between the alleles at either exon 4 or 10. The three *vasa* transcripts are most probably alternative splice variants from a single *vasa* gene, and the alleles are not heterozygous. The *C. semilaevis* genome has been published [56]. Comparison of our results with the published confirmed that only one *vasa* gene locus exists from which the three variants originate.

qRT–PCR analysis of the tissue distribution pattern of *vasa* implied that *vas-l* and *vas-m* were exclusively expressed in the gonads. The gonad-specific expression pattern of *vasa* is in agreement with the function of *vasa* as a germline marker and is similar to that found in other teleosts [11], [14]–[18], [57]–[61]. The results show that despite the dominant *vas-s* expression in the gonads, a very weak expression of this gene was also detected in other tissues. A very low expression of *vasa* in the extragonadal tissues has also been reported in other teleosts, such as rainbow trout [11], European sea bass [12], and Senegalese sole [13]. These expressions are attributed to the helicase activity of VASA protein. This protein is a possible translational regulator of certain mRNAs, which is essential to the specification and differentiation of germ cells and somatic cells [62]–[65]. The relative expression level of the *vasa* transcript was much higher in the ovary compared with that in the testis, especially the *vas-s*. Differential expression of *vasa* variants between males and females has also been described. In Senegalese sole, the long *Ssvasa1* and *Ssvasa2* are expressed in both sexes, whereas very low expression of *Ssvasa3* and *Ssvasa4* are detected in adult males [13]. In tilapia, the expression of the short *vas-s* isoform is predominant compared with *vas* expression in the ovary. By contrast, the expression of the long *vas* isoform is predominant in the testis [16]. Zebrafish only contain the short variant *vas-δ4* in adult males, whereas both the long *vas-l* and *vas-δ4* are expressed in adult females [19].

ISH during oogenesis and spermatogenesis revealed the strong signals of *vasa* mRNA in the spermatocytes as well as oocytes and weak signals in the spermatogonia. No signal was detected in somatic cells for both ovary and testis. The results suggested the expression and distribution patterns of *vasa* mRNA in male and female germ cells during gametogenesis. The *vasa* gene can thus be considered as a germline-specific marker in *C. semilaevis*.

The expression patterns of the three *vasa* transcripts during embryonic development were all similar and also had the same tendency variation on the whole. The relative expression levels of these transcripts also resemble those in the gonads. The three *vasa* transcripts were present in unfertilized eggs, strongly suggesting that these transcripts are maternally inherited. The relative expression of *vasa* mRNAs was kept stable during the first two stages compared with that in the unfertilized eggs. We inferred that no zygotic transcripts and decays exist in the early embryos and the amounts of the maternally inherited mRNAs (e.g., *vasa*) changed slightly. Recently, genome-wide analysis of the maternal-to-zygotic transition (MZT) has been conducted in *Drosophila* and zebrafish [66]–[71]. Based on these studies, the MZT in somatic cells and those in PGCs were marked in our results (Figure 5). The somatic MZT caused the massive degradation of maternal mRNAs and the zygotic genome activation in the soma. The MZT in PGCs was delayed relative to that in the soma. Studies on turbot (*Scophthalmus maximus*; *Pleuronectiforme*) have revealed that *Smvas* localized at cleavage furrows during embryogenesis resemble the pattern observed in zebrafish rather than medaka [14], [72], [73]. Therefore, the PGCs of *C. semilaevis* are probably specified by the preformation mode and inherit maternal germ plasms (e.g., *vasa*, *nanos*, and *piwi*). Thus, we hypothesized that the amount of *vasa* mRNAs localized in PGCs continued its stability with the decay of other maternal mRNAs and the rapid zygotic transcription of somatic mRNAs during the somatic MZT. This hypothesis may explain the initial increase in the relative expression level, the reduction from 8-cell stage to 16-cell stage, and the continuous decrease until the morula stage. After the morula stage, *vasa* expression began to increase and quickly reached the peak value at early gastrula stage. The MZT occurred in the PGCs during these stages, which resulted in the decay of maternal mRNAs and activated the massive transcription of zygotic mRNAs in the PGCs along with the proliferation of PGCs. The amount of newly transcribed zygotic *vasa* transcripts probably increased much faster than the decay of maternal ones. This result facilitated the identification of a period of observing GFP if microinjection of the GFP expression vector driven by *vasa* promoter is performed. A sharp decrease in the *vasa* expression level was found in the following two stages, and *vasa* expression remained at this level during the last few stages. In zebrafish [36], gibel carp [57], and Japanese flounder [18], the high *vasa* expression level also dramatically declined following the early gastrula stage. This reduction is attributed to the dilution of the *vasa* mRNAs and the PGCs within a growing embryo.

The profiles of *vasa* expression levels during gonadal differentiation have been examined in European sea bass [12] and Senegalese sole [13]. Discriminating females from males just based only on the phenotype with the indistinguishable primitive gonads during the early gonadal differentiation is difficult. Given this technical limit, the samples used during this stage were mixed groups in previous studies. In this study, heterogametic females (ZW) were identified by the female-specific PCR product from their primers [33]. The juveniles were cultivated under 23°C to avoid sex reversal induced by high temperature. Considering that *C. semilaevis* females grow faster than males, we chose juveniles based on their size. Similar to the studies in European sea bass [12], Senegalese sole [13], and winter flounder [74], we proposed that the TL of the juveniles has more pronounced effect on gonadal differentiation than the teleost age. qRT–PCR results indicated that only the *vas-s* transcript can be detected during early gonadal differentiation. This result suggested a shift in the expression of *vasa* transcripts after the localization of PGCs to gonadal anlagen and the mediation by the interactions with the surrounding somatic cells during gonadal differentiation. The *vas-s* expression level in females decreased at TL = 40 mm. Histological observation revealed that a portion of the PGCs started to enter into mitosis and form clusters of germ cells at this stage [75]. By contrast, the *vas-s* expression increased in males at TL = 50 mm, at which the testis entered into mitosis and gonadal differentiation [75]. The sexual discrepancy are attributed to the opposite expression trends of *vas-s* expression before PGCs enter into mitosis. We speculate that the two stages have vital functions in the formation of germ cells and sexual differentiation. PGCs are probably more susceptible to the external environment during these stages, which sheds new light on the production of infertile or sterile fish. In our further studies, we will focus on the function of *vasa* in the gonadal differentiation or how this process regulates the expression patterns of *vasa*.

## Conclusions

We reported the full length of three *vasa* cDNA sequences and genomic DNA sequence in *C. semilaevis*. The *vasa* genomic organization and structural differences between the three *vasa* transcripts were demonstrated. The expression profiles of *vasa* transcripts confirmed the use of *vasa* as a molecular marker for the germ cell linage in *C. semilaevis*. This study lays the foundation for constructing GFP expression vectors driven by the *vasa* to label, isolate, and transplant the PGCs of this species. This study also improves the development of a genetic breeding technique for *C. semilaevis*.

## Supporting Information

Figure S1 **Sequence alignments of the three** ***vasa*** **isoforms.** Nucleotides were numbered to the right. The nucleotides in light and dark grey backgrounds implied the *vas-l* and *vas-m* unique sequences. The polyadenylation signal and the poly-A tail were marked in boldface. The asterisk indicated stop codon.
(TIF)

Figure S2 **PCR products containing exons 4 and 10.** Using specific primers ex4-FW/RV and ex10-FW/RV as well as genomic DNA as templates, only a single PCR product contained exon 4 or 10 in males and females.
(TIF)

Table S1 **Sequences of primers used for cloning and expression analysis of** ***vasa.***
(PDF)
