# Supplementary figures and images for: Sexually Dimorphic Expression of vasa Isoforms in the Tongue Sole (Cynoglossus semilaevis)
Source: PLoS One. 2014 Mar 26;9(3):e93380. doi: 10.1371/journal.pone.0093380 (PMC3966880; doi:10.1371/journal.pone.0093380)

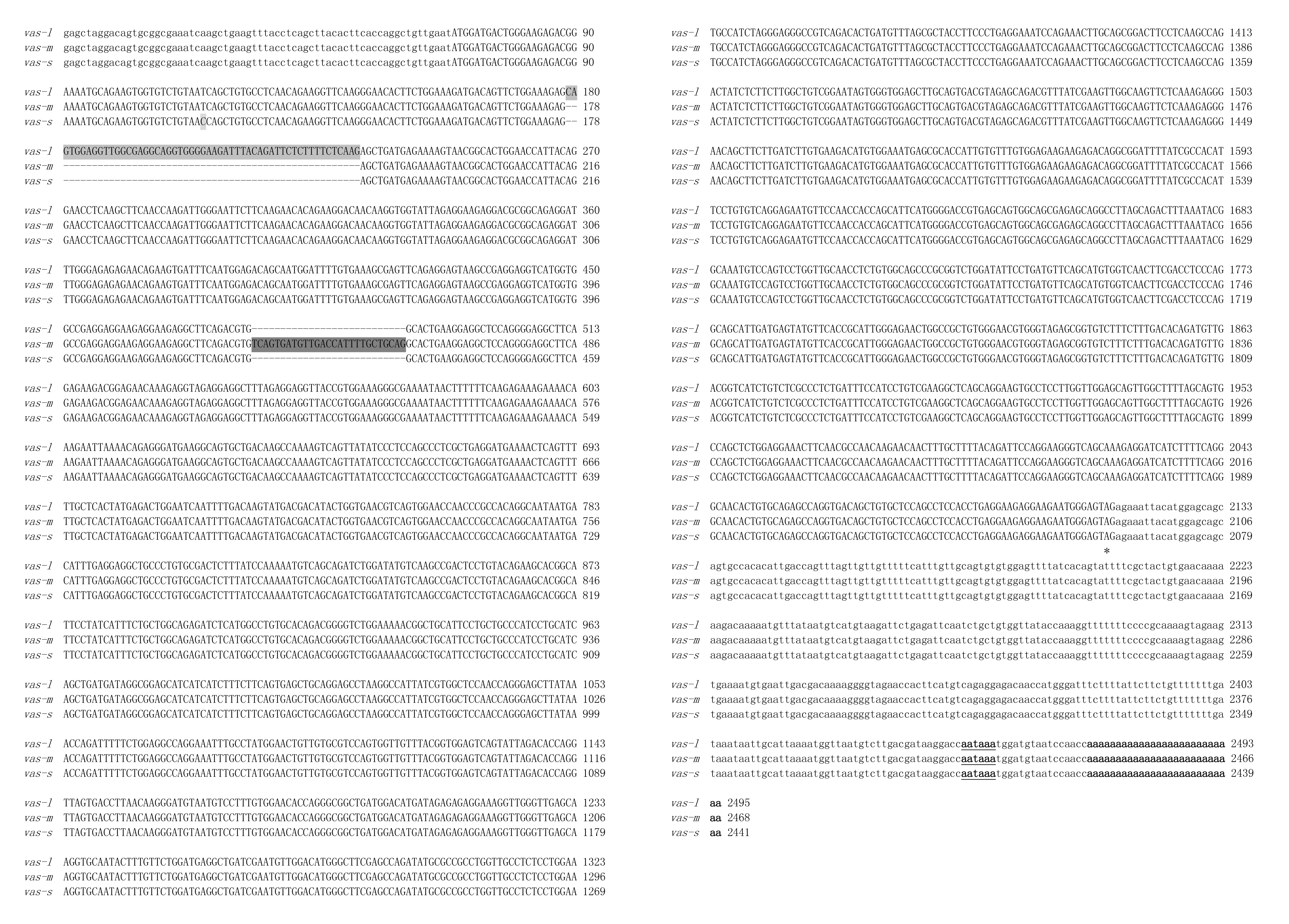

Supplement: Figure S1 — Sequence alignments of the three vasa isoforms. Nucleotides were numbered to the right. The nucleotides in light and dark grey backgrounds implied the vas-l and vas-m unique sequences. The polyadenylation signal and the poly-A tail were marked in boldface. The asterisk indicated stop codon. (TIF) [file pone.0093380.s001.tif]

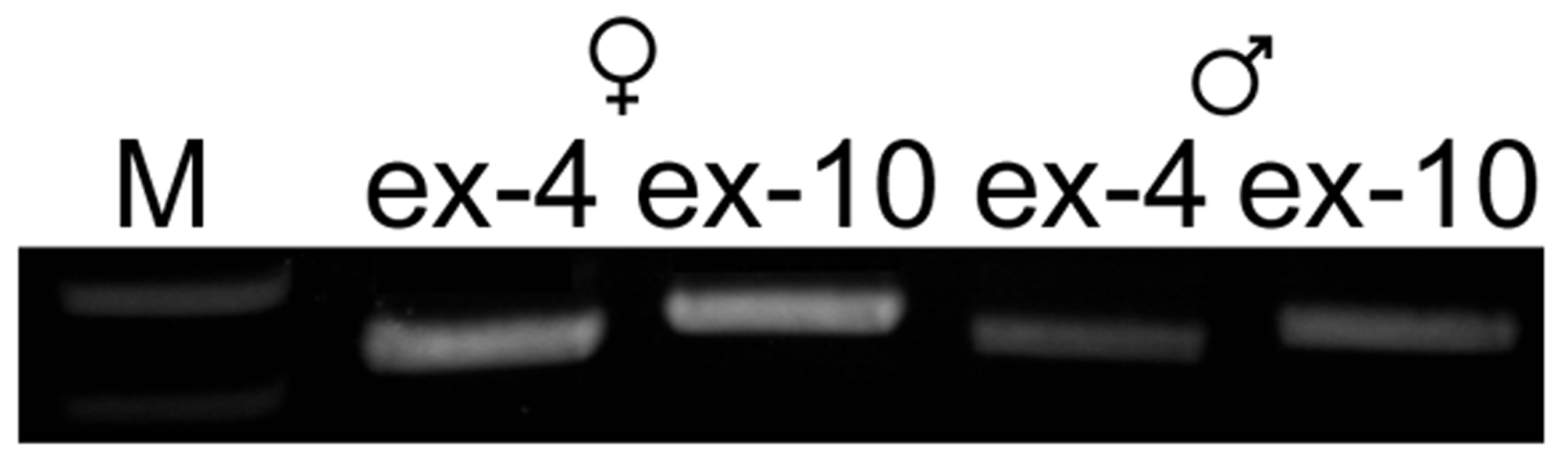

Supplement: Figure S2 — PCR products containing exons 4 and 10. Using specific primers ex4-FW/RV and ex10-FW/RV as well as genomic DNA as templates, only a single PCR product contained exon 4 or 10 in males and females. (TIF) [file pone.0093380.s002.tif]
