# Supplementary material for: Sexually Dimorphic Expression of vasa Isoforms in the Tongue Sole (Cynoglossus semilaevis)
Source: PLoS One. 2014 Mar 26;9(3):e93380. doi: 10.1371/journal.pone.0093380 (PMC3966880; doi:10.1371/journal.pone.0093380)
Supplement: Table S1 — Sequences of primers used for cloning and expression analysis of vasa. (PDF) [file pone.0093380.s003.pdf]

Table S1**. Sequences of primers used for cloning and expression analysis of *vasa***

| Primers | Sequence(5′—3′) | Usage |
|---|---|---|
| scaf-FW | GGCGGAGCATCATCATCTTTC | RT–PCR |
| scaf-RV | TACTCATCAATGCTGCCTGGG | RT–PCR |
| 5′race1 | CCCACTATTCCGACAGCCAAG | 5′RACE |
| 5′race2 | GGACGCACAACAGTTCCATAGG | 5′RACE |
| 3′race1 | TAAATACGGCAAATGTCCAGTCC | 3′RACE |
| 3′race2 | TCACCGCATTGGGAGAACTGG | 3′RACE |
| vasa-5′FW | GGACAGTGCGGCGAAATCAAG | LA PCR |
| vasa-3′RV | GGTCCTTATCGTCAAGACATTAACC | LA PCR |
| 5′GSP1 | ACATAAGGTCCTCTGGTGGATTGG | Genome Walking |
| 5′GSP2 | CGCTTTCTGCTGAGCTTTCCATA | Genome Walking |
| 5′GSP3 | GACATGATGTTGCCATGACTGAAAC | Genome Walking |
| 3′GSP1 | GTTGCAGTGTGTGGAGTTTTATCACAG | Genome Walking |
| 3′GSP2 | GGTAAAACCACTTCATGTCAGAGGAG | Genome Walking |
| 3′GSP3 | GGTTAATGTCTTGACGATAAGGACCAA | Genome Walking |
| SP6-FW | ATTTAGGTGACACTATAGAAGCGAATGG<br>GAGTAGAGAAATTACATGGA | Probe Synthesis |
| T7-RV | TAATACGACTCACTATAGGGAGA<br>GTTGGATTACATCCATTTATTGGTC | Probe Synthesis |
| ex4-FW | TATGGATGACTGGGAAGAGACGGTA | PCR |
| ex4-RV | CTGTCCATGCAGGTCTTATACTAGTGAT | PCR |
| ex10-FW | TGGGAGAGAGAACAGAAGTGATTTCAA | PCR |
| ex10-RV | CACTGGTTATCATCGTCACTTTTATATGG | PCR |
| vasaL-FW | TGGGAAGAGACGGAAAATGC | qRT–PCR |
| vasaL-RV | GTAAATCTTCCCCACCTGCC | qRT–PCR |
| vasaM-FW | CAATGGATTTTGTGAAAGCGA | qRT–PCR |
| vasaM-RV | GCAGCAAAATGGTCAACATCA | qRT–PCR |
| vasaS-FW | TGGAAAGAGAGCTGATGAGAAAAG | qRT–PCR |
| vasaS-RV | CCTCCTTCAGTGCCACGTC | qRT–PCR |
| 18S-FW | GGTAACGGGGAATCAGGGT | qRT–PCR |
| 18S-RV | TGCCTTCCTTGGATGTGGT | qRT–PCR |
| B2M-FW | TGTTCGTCGTTCTGCCGTGT | qRT–PCR |
| B2M-RV | TCAGGGTGTTGGGCTTGTTGT | qRT–PCR |
| RPL17-FW | AGTGCGTCCCGTTCCGTC | qRT–PCR |
| RPL17-RV | TCAGCGTTGCTCTCTGCGTT | qRT–PCR |
